# Peripartum Hysterectomies over a Fifteen-year Period

**DOI:** 10.1055/s-0040-1721354

**Published:** 2021-01-29

**Authors:** Alessandra Dorigon, Sérgio Hofmeister Martins-Costa, José Geraldo Lopes Ramos

**Affiliations:** 1 Hospital de Clínicas de Porto Alegre, Porto Alegre, RS, Brazil; 2 Department of Gynecology and Obstetrics, Faculty of Medicine, Universidade Federal do Rio Grande do Sul, Porto Alegre, RS, Brazil

**Keywords:** puerperal hysterectomy, postpartum hemorrhage, placenta accreta, placenta previa, histerectomia puerperal, hemorragia pós-parto, placenta acreta, placenta prévia

## Abstract

**Objective** To determine the indications and outcomes of peripartum hysterectomies performed at Hospital de Clínicas de Porto Alegre (a university hospital in Southern Brazil) during the past 15 years, and to analyze the clinical characteristics of the women submitted to this procedure.

**Methods** A cross-sectional study of 47 peripartum hysterectomies from 2005 to 2019.

**Results** The peripartum hysterectomies performed in our hospital were indicated mainly due to placenta accreta or suspicion thereof (44.7% of the cases), puerperal hemorrhage without placenta accreta (27.7%), and infection (25.5%). Total hysterectomies accounted for 63.8% of the cases, and we found no difference between total versus subtotal hysterectomies in the studied outcomes. Most hysterectomies were performed within 24 hours after delivery, and they were associated with placenta accreta, placenta previa, and older maternal age.

**Conclusion** Most (66.0%) patients were admitted to the intensive care unit (ICU). Those who did not need it were significantly older, and had more placenta accreta, placenta previa, or previous Cesarean delivery.

## Resumo

**Objetivo** Determinar as indicações e os desfechos das histerectomias periparto realizadas no Hospital de Clínicas de Porto Alegre nos últimos 15 anos, bem como analisar as características clínicas das mulheres submetidas a esse procedimento.

**Métodos** Estudo transversal de 47 histerectomias periparto realizadas no período de 2005 a 2019.

**Resultados** Em nosso hospital, as histerectomias periparto foram indicadas principalmente por acretismo placentário ou sua suspeita (44,7% dos casos), hemorragia puerperal sem acretismo placentário (27,7%), e infecção (25,5%). Histerectomias totais corresponderam a 63,8% dos casos, e não encontramos diferença entre histerectomia total e subtotal para os desfechos estudados. A maioria das histerectomias foi realizada dentro de 24 horas após o parto, o que estava associado a acretismo placentário, placenta prévia, e idade materna mais avançada.

**Conclusão** A maioria (66,0%) das mulheres necessitou de internação em unidade de terapia intensiva (UTI); aquelas que não necessitaram eram significativamente mais velhas, e tinham mais acretismo placentário, placenta prévia, ou cesárea prévia.

## Introduction

In the history of Obstetrics, Horatio Storer performed the first hysterectomy on a gravid uterus in a live woman in 1869 due to a hemorrhage caused by a uterine tumor. 1 The role of peripartum hysterectomy has changed ever since. In the 20th century, it was even indicated as a procedure for sterilization. 2

Currently, peripartum hysterectomy is performed mainly in cases of abnormal placentation (predominantly placenta accreta) or life-threatening hemorrhage. 3 4 5 A meta-analysis 3 of 128 studies from low- to high-income countries estimated a weighted average of 0.9 peripartum hysterectomies per 1,000 births, and a maternal mortality rate of 5.2% after this procedure.

Both primary or repeat Cesarean sections are significantly associated with an increased risk of peripartum hysterectomy, not only due to abnormal placentation, but also due to uterine atony. 6 7 8 9 Other risk factors are placenta previa (which is significantly associated with an increased likelihood of placenta accreta), advanced maternal age, twin pregnancy, chorioamnionitis, and puerperal infections. 3 7 8 10 11 12

Women undergoing peripartum hysterectomy are at risk of postoperative complications, such as admission to the intensive care unit (ICU), blood transfusion, bladder and ureteral injury, reoperation, and wound complications. 3 5 13 14 15

The objective of the present study was to analyze the peripartum hysterectomies performed at Hospital de Clínicas de Porto Alegre in the past 15 years to provide an evaluation of their main indications at our hospital. We also aimed to analyze the clinical characteristics of the patients submitted to this procedure.

## Methods

The present cross-sectional study used retrospective data from the electronic health records stored in the database of Hospital de Clínicas de Porto Alegre from January 2005 (when the electronic health records were effectively implemented in our hospital) until August 2019 (final date of the search data). All 47 hysterectomies performed during this period due to pregnancy or puerperal complications were included. We chose to analyze some obstetric near-miss factors related to hysterectomy indications: hysterectomy time (up to 24 hours), admission to the ICU, use of antibiotics, type of hysterectomy (subtotal or total), and need of red-blood-cell transfusion. The prophylactic use of antibiotics was not considered, as it is part of the Cesarean-section protocol. Only the use of antibiotics in the postpartum period was considered. The statistical analysis was performed using the Statistical Package for the Social Sciences (SPSS, IBM Corp., Armonk, NY, US) software. The Student *t* -test was used for the continuous variables, and the Fisher exact test, for the categorical variables. Values of *p* < 0.05 were considered statistically significant. The present study was registered at Plataforma Brasil, the Brazilian unified database of research records involving human beings. Ethics approval was granted by the Ethics Committee of Hospital de Clínicas de Porto Alegre. The authors signed a data-confidentiality agreement.

## Results

The present study identified 47 cases of hysterectomies performed due to complications during pregnancy or the puerperium in the period from 2005 to 2019 in our hospital. Considering that 54,617 births occurred in this period, we estimate a rate of 0.87 peripartum hysterectomies per 1,000 births.

Maternal characteristics and previous and current pregnancy complications are shown in Table 1 . The mean maternal age was 31.2 years, the mean BMI was of 30.9 kg/m ^2^ , and 59.6% of the patients had a previous Cesarean section. In the current pregnancy, the most frequent complications were intrauterine infection and/or puerperal infection and placenta accreta (44.7% and 42.6% of the cases respectively). Placenta previa was present in 27.7% of the patients.

**Table 1:** Maternal characteristics and previous and current pregnancy complications

Maternal characteristics and previous and current pregnancy complications	n	Mean (standard deviation) or % (n)
Maternal age (years)	47	31.2 (6.3)
Previous cesarean section	47	59.6% (28)
Maternal weight (kg)	46	81.1 (16.3)
Maternal Body Mass Index (kg/m ^2^ )	46	30.9 (6.0)
History of previous postpartum hemorrhage	47	8.5% (4)
Placenta previa in the current pregnancy	47	27.7% (13)
Placenta accreta in the current pregnancy	47	42.6% (20)
Preterm labor in the current pregnancy	47	19.1% (9)
Prelabor rupture of membranes in the current pregnancy	47	10.6% (5)
Cephalopelvic disproportion in the current pregnancy	47	8.5% (4)
Postpartum hemorrhage in the current pregnancy	47	55.3% (26)
Intrauterine infection and/or puerperal infection	47	44.7% (21)
Urinary tract infection in the current pregnancy	47	29.8% (14)

Table 2 shows the indications for Cesarean section and peripartum hysterectomy and the maternal and fetal outcomes. In total, 74.5% of the deliveries were by Cesarean section, and its main indication was placenta previa. The most frequent cause of hysterectomy was placenta accreta or suspicion thereof (44.7% of peripartum hysterectomies), followed by postpartum hemorrhage without placenta accreta or suspicion (27.7%) and puerperal infection (25.5%). In addition, 63.8% of the hysterectomies were total, and 66.0% were performed within 24 hours after delivery. Most patients required red-blood-cell transfusion and admission to the ICU (76.6% and 66.0% respectively). The mean gestational age at delivery was 37.2 weeks.

**Table 2:** Indications for Cesarean delivery and for peripartum hysterectomy and maternal and fetal outcomes

Indications for Cesarean delivery and for peripartum hysterectomy and maternal and fetal outcomes	n	Mean (standard deviation) or % (n)
Cesarean delivery in the current pregnancy	47	74.5% (35)
Indication for cesarean delivery		
Placenta previa	35	37.1% (13)
Fetal distress	35	17.1% (6)
Cephalopelvic disproportion	35	11.4% (4)
Placenta accreta or suspicion thereof with no placenta previa	35	11.4% (4)
Cause of peripartum hysterectomy		
Placenta accreta or suspicion thereof	47	44.7% (21)
Postpartum hemorrhage with no placenta accreta nor suspicion thereof	47	27.7% (13)
Puerperal infection and suspicion thereof	47	25.5% (12)
Uterine rupture	47	2.1% (1)
Total hysterectomy	47	63.8% (30)
Hysterectomy within 24 hours after delivery	47	66.0% (31)
Antibiotic therapy during delivery or hysterectomy hospitalizations or in the postpartum period	47	59.6% (28)
Transfusion of red blood cells	47	76.6% (36)
Intensive care admission	47	66.0% (31)
Gestational age at delivery (weeks)	47	37.2 (3.2)
Male newborn	45	35.6% (16)
Birth weight (kg)	45	2.876 (0.686)

**Fig. 1:**
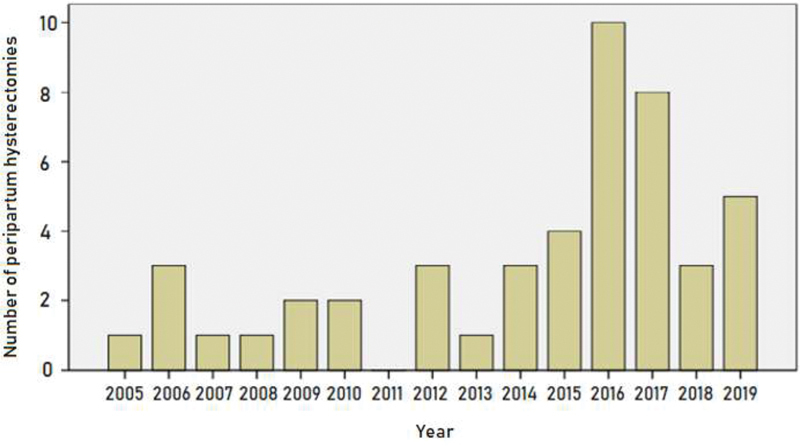
Timeline of peripartum hysterectomies.

Fig. 1 shows the annual distribution of peripartum hysterectomies in our hospital from 2005 to 2019. There was a peak in the number of hysterectomies in 2016 and 2017, and a subsequent reduction in 2018.

Table 3 compares peripartum hysterectomy within 24 hours after delivery with hysterectomy performed more than 24 hours after delivery. Earlier hysterectomy was associated with older maternal age (33.3 versus 27.1 years; *p* = 0.001), placenta accreta (54.8% versus 18.8%; *p* = 0.029), and placenta previa (38.7% versus 6.3%; *p* = 0.036). Premature rupture of membranes was less common in cases of earlier peripartum hysterectomy (3.2% versus 25.0%; *p* = 0.040).

**Table 3:** Peripartum hysterectomy within 24 hours after delivery

	Hysterectomy after 24h after delivery		Hysterectomy within 24h after delivery		*p-value*
	n	Mean (standard deviation) or % (n)	n	Mean (standard deviation) or % (n)	
Previous Cesarean section	16	43.8% (7)	31	67.7% (21)	0.131
History of previous postpartum hemorrhage	16	0% (0)	31	12.9% (4)	0.284
Placenta previa	16	6.3% (1)	31	38.7% (12)	0.036
Placenta accreta	16	18.8% (3)	31	54.8% (17)	0.029
Prelabor rupture of membranes	16	25.0% (4)	31	3.2% (1)	0.040
Preterm labor	16	31.3% (5)	31	12.9% (4)	0.239
Cephalopelvic disproportion	16	12.5% (2)	31	6.5% (2)	0.597
Maternal age (years)	16	27.1 (6.6)	31	33.3 (5.1)	0.001
Maternal Body Mass Index (kg/m ^2^ )	15	30.5 (6.2)	31	31.1 (6.0)	0.742
Gestational age at delivery (weeks)	16	37.3 (3.7)	31	37.1 (3.0)	0.835
Birth weight (kg)	14	3.068 (0.768)	31	2.790 (0.640)	0.212

Table 4 shows that patients who were not admitted to the ICU were significantly older (33.3 versus 30.1 years; *p* = 0.045), had a higher prevalence of placenta accreta (62.5% versus 32.3%; *p* =. 065), of placenta previa (50.0% versus 16.1%; *p* = 0.020), and of previous Cesarean section (87.5% versus 45.2%; *p* = 0.006).

**Table 4:** Admission to the intensive care unit (ICU)

	NO ICU ADMISSION		ICU ADMISSION		*p-value*
	n	Mean (standard deviation) or % (n)	n	Mean (standard deviation) or % (n)	
Previous Cesarean section	16	87.5% (14)	31	45.2% (14)	0.006
History of previous postpartum hemorrhage	16	12.5% (2)	31	6.5% (2)	0.597
Placenta previa	16	50.0% (8)	31	16.1% (5)	0.020
Placenta accreta	16	62.5% (10)	31	32.3% (10)	0.065
Prelabor rupture of membranes	16	12.5% (2)	31	9.7% (3)	1.000
Preterm labor	16	18.8% (3)	31	19.4% (6)	1.000
Cephalopelvic disproportion	16	0% (0)	31	12.9% (4)	0.284
Maternal age (years)	16	33.3 (3.5)	31	30.1 (7.2)	0.045
Maternal Body Mass Index (kg/m ^2^ )	16	30.8 (6.9)	30	31.0 (5.6)	0.894
Gestational age at delivery (weeks)	16	36.8 (2.5)	31	37.4 (3.6)	0.595
Birth weight (kg)	16	2.650 (0.493)	29	3.001 (0.751)	0.101

We compared the use of postpartum antibiotics during the hospitalization with patients who did not use antibiotic therapy. We can see that the women treated with antibiotics were younger (29.5 versus 33.7 years; *p* = 0.024) and had preterm labor more frequently, but that was not statistically significant (28.6% versus 5.3%; *p* = 0.064). We found no statistically significant difference between total and subtotal hysterectomy for the clinical factors analyzed. Blood transfusion was not associated with any of these factors either.

## Discussion

In the present study, we found that the rate of peripartum hysterectomies was of 0.87 per 1,000 births. This number is very similar to the weighted average peripartum hysterectomy of 0.9 per 1,000 births provided by the aforementioned meta-analysis, 3 although still above many estimates from high-income countries. 3 4 5

The main cause of peripartum hysterectomy in the present study – placenta accreta or suspicion thereof – was responsible for 44.7% of the cases. Although our hospital is a referral center for these patients, this finding is consistent with systematic reviews that report placental abnormalities as the most common indication, followed by uterine atony and other causes, such as uterine rupture and infection (including chorioamnionitis and endometritis). 3 4 5 However, the fact that 25.5% of our peripartum hysterectomies are due to infection contrasts with most other series, in which it accounts for 2% or less of hysterectomies. 3 4 5 16

In the present study, 74.5% of deliveries were Cesarean sections, and their main indication was placenta previa. This may be related both to the increased risk of peripartum hysterectomy after primary or repeat Cesarean section, and to the strong association between placenta previa and placenta accreta. 6 7 8 9 11 12 At Hospital de Clínicas de Porto Alegre, the rate of Cesarean section in the same period was of 34.2%.

Total hysterectomy represented 63.8% of the hysterectomies performed in our patients. The proportion of total versus subtotal hysterectomies varies in different studies. Two systematic reviews reported rates of 51.1% 3 and 52.2% 5 of total hysterectomies, but large series 14 have also reported higher proportions. In addition, in the present study there was no significant difference between total and subtotal hysterectomy for the analyzed outcomes. The comparison between the two techniques produced controversial results, and, while some series of studies found statistically significant differences 14 , others did not. 13 17

Blood transfusion and ICU admission were necessary for 76.6% and 66.0% of the patients respectively. According to systematic reviews and other studies, many patients undergoing peripartum hysterectomy require blood transfusion and ICU admission. 3 5 13 14 15

We observed that the use of antibiotic therapy during the hospitalization was associated with premature labor, which was 5.4 times more frequent in women treated with antibiotics (28.6% versus 5.3%), although the *p* -value was not statistically significant (0.064). Inflammation and infection are part of the pathogenesis of premature labor, which possibly explains a greater need for antibiotics in this group of patients. Another discussion point would be if the use of antepartum antibiotic therapy could select infecting bacteria in the postpartum period.

The finding that hysterectomy more than 24 hours after birth was associated with premature rupture of membranes may have been biased by a small number of cases in each group (4 after 24 hours versus 1 within 24 hours). On the other hand, the association of hysterectomy performed in the first 24 hours after delivery with placenta accreta, placenta previa and more advanced maternal age is expected. Many cases of placenta accreta in the present series were suspected before birth, leading to early intervention. Some were even preprogrammed.

In addition, women in the present series who were not admitted to the ICU were also significantly older and more likely to have placenta previa or undergo Cesarean section. Although the difference was not statistically significant ( *p* = 0.065), 62.5% of these patients had placenta accreta versus 32.3% of those who were admitted to the ICU. The prenatal suspicion of cases of placenta accreta in the present series enabled the adoption of special measures, possibly reducing maternal morbidity. When placenta accreta is suspected, the care of a multidisciplinary team and not attempting placenta removal both improve the results. 18 19 20 In addition, placenta previa, advanced maternal age and Cesarean section are associated with placenta accreta. 11 12

There were two maternal deaths in the present series, one due to hemorrhagic shock and the other due to septic shock. The mortality rate for peripartum hysterectomies was of 4.3%, which is lower than the 5.2% estimate of a meta-analysis 3 of 128 studies from different countries (11.9% in low- and middle-to-low-income countries, compared with 2.5% in high- and middle-to-high-income countries).

## Conclusion

We found an expressive association between peripartum hysterectomy and placenta previa and placenta accreta, which was an important factor in the indication of hysterectomy. This situation has been advancing in Brazil in recent years because of the excessive performance of Cesarean sections.
